# Standardization of labor induction management: A mixed‐methods evaluation of the clinician perspective

**DOI:** 10.1002/pmf2.70117

**Published:** 2025-09-26

**Authors:** Jessica K. Wu, Rinad S. Beidas, Julia E. Szymczak, Sindhu K. Srinivas, Samuel Parry, Lisa D. Levine, Rebecca F. Hamm

**Affiliations:** ^1^ Department of Obstetrics & Gynecology University of Pennsylvania Perelman School of Medicine Philadelphia Pennsylvania USA; ^2^ Department of Medical Social Sciences Northwestern University Feinberg School of Medicine Chicago Illinois USA; ^3^ Department of Internal Medicine University of Utah Spencer Fox Eccles School of Medicine Salt Lake City Utah USA; ^4^ Pregnancy and Perinatal Research Center Division of Maternal‐Fetal Medicine Department of Obstetrics & Gynecology University of Pennsylvania Perelman School of Medicine Philadelphia Pennsylvania USA; ^5^ Leonard Davis Institute of Health Economics University of Pennsylvania Perelman School of Medicine Philadelphia Pennsylvania USA

**Keywords:** clinician perspective, labor induction, mixed‐methods, standardization

## Abstract

**Introduction:**

Standardizing labor induction (IOL) practices may reduce cesarean delivery rates and improve obstetric disparities. Such labor induction protocols focus on active management with recommendations for frequent cervical exams, early amniotomy, and intervention when no cervical change is made (e.g., oxytocin or intrauterine pressure catheter). Here, we aimed to characterize the clinician perspective on the feasibility and acceptability of each of these components of a standardized labor induction protocol to improve implementation of standardized labor induction practices.

**Methods:**

This sequential mixed‐methods study first surveyed clinicians after implementation of a standardized labor induction protocol at two sites in 2021. We used the four‐question validated Acceptability of Intervention Measure (AIM; total 4–20) as well as a single acceptability item (1–5) for each of eight specific protocol components. Total AIM scores were grouped into tertiles. Clinicians in the top (“High” Acceptability) and bottom (“Low” Acceptability) tertiles were purposively sampled to participate in a semi‐structured interview. Topics included (1) acceptability of protocol components, (2) barriers/facilitators to adherence, and (3) strategies for overcoming barriers. Interviews were coded using an integrated approach with high inter‐rater reliability (*k* = 0.83).

**Results:**

A total of 104 clinicians (57 physicians, 43 RNs, 4 CNMs) completed the survey. Median total AIM score was 15/20 [IQR 12–19]. Protocol components with the highest scores on the individual acceptability item focused on not continuing “futile” cervical ripening, as well as interventions for active labor dystocia. Components with low acceptability included frequent cervical exams and early amniotomy. A total of 24 clinicians were interviewed (12 “High” and 12 “Low” Acceptability). Clinicians described barriers to implementation of less acceptable components of the protocol, including staff/time constraints, best‐practice disagreements, and concerns around patient satisfaction. Clinicians recommended more education around the protocol's safety/efficacy and communication about anticipated labor induction steps to overcome these barriers.

**Conclusion:**

This work describes the clinician perspective on specific labor induction interventions and provides concrete recommendations to overcome implementation barriers of a standardized labor induction protocol.

## 1 INTRODUCTION

Induction of labor has increased in the United States, with rates increasing from 9.6% in 1990 to 31.9% of all births in 2022 [1]. As a part of this shift, standardization of labor induction practices using evidence‐based active management has been shown to play a role in improving obstetric outcomes, as well as reducing racial disparities in those outcomes. Specifically, studies have demonstrated associations between standardized induction protocols and reductions in labor length, infectious morbidity, postpartum hemorrhage, and composite maternal and neonatal morbidity [2, 3, 4, 5]. Standardization of labor induction has also been associated with reduced racial disparities in the cesarean rate and increased satisfaction with the birth experience [6, 7].

However, there remains wide variation in labor induction management, with components of labor induction decision‐making such as frequency of cervical exams, utilization of artificial membrane rupture, oxytocin, intrauterine pressure catheters (IUPCs), and thresholds for cesarean delivery differing significantly across and within centers. Prior study has identified that specific labor interventions, such as frequent cervical exams in latent and active labor, are associated with reduced cesarean rates and neonatal morbidity [8]. While some prior work has assessed clinician perspectives on labor induction and its practices in general [9, 10], a labor induction protocol is ultimately the implementation of multiple components, each with its own unique challenges. It is therefore critical to understand the barriers and facilitators to implementing each component of an induction protocol in a clinical setting.

Here, we aimed to expand on prior work by our team describing clinicians’ general acceptability of a standardized labor induction protocol [10] to specifically characterize and compare the clinician perspective on the feasibility and acceptability of each protocol component using both quantitative and qualitative methods, with the goal of harnessing these data for future implementation success across labor units.

## 2 MATERIALS AND METHODS

This sequential mixed‐methods study characterized clinician perspectives after the implementation of a standardized induction of labor protocol at two urban labor and delivery sites within the University of Pennsylvania Health System in 2021. All study procedures were approved by the University of Pennsylvania Institutional Review Board; informed consent was obtained prior to initiation of survey and interview components of the study. Standards for Reporting Qualitative Research (SRQR) guidelines were utilized in preparing this manuscript, which represent validated best practices for presentation of qualitative work such as appropriate description and justification of research methods and effective reporting of qualitative results [11].

**FIGURE 1:**
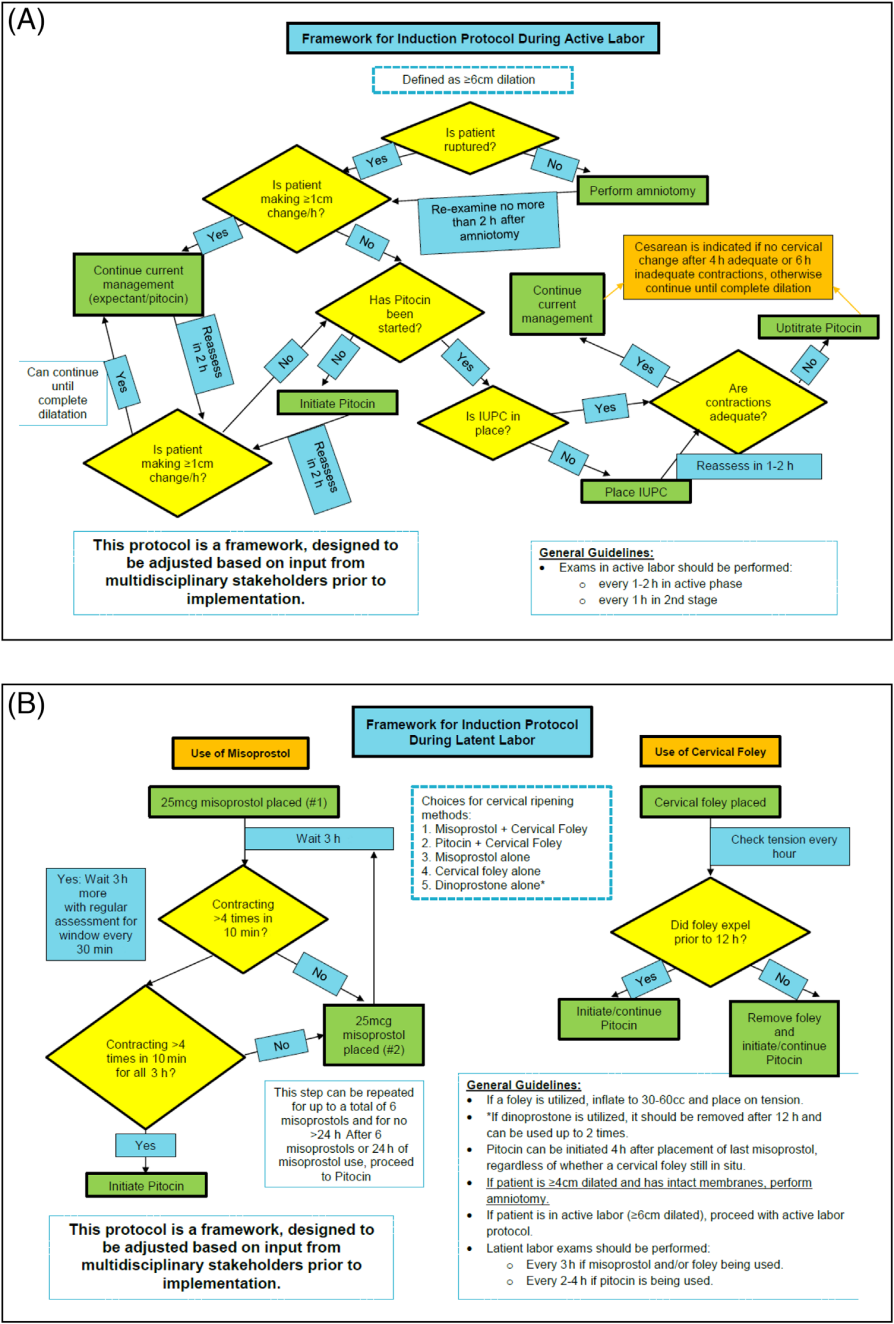
(A) Standardized labor induction protocol for latent labor. (B) Standardized labor induction protocol for active labor.

This work was embedded into an overarching hybrid 2‐year pre‐ and post‐implementation trial, beginning October 1, 2018 and concluding December 31, 2022. Additional details on study design and clinical outcomes from the overarching study were previously published [3, 10]. In short, a standardized labor induction protocol was approved by a multidisciplinary institutional obstetrical committee prior to its initiation at each of two labor units, including recommendations for frequent cervical exams and interventions such as oxytocin and amniotomy, with a focus on active management of labor induction (Figure 1). The protocol was initiated at Site #1 on October 1, 2020, and at Site #2 on January 1, 2021.  Implementation of this induction protocol was associated with reduced maternal morbidity, without impacting cesarean delivery rate [10].

Six months after implementation, a survey was distributed to clinicians, including the four‐item Acceptability of Intervention Measure (AIM) and a single‐acceptability item (Likert scale 1–5; ranging from “not at all comfortable” to “completely comfortable”) about each of eight specific protocol components. The AIM is a validated measure with strong psychometric properties commonly used to assess acceptability across health care interventions [12, 13, 14]. A selection of 4 or 5 on the Likert scale for each component was deemed “comfortable” with that component.

Clinicians who were classified as having “High” and “Low” acceptability based on AIM scores were then approached to participate in a semi‐structured interview. The interview guide was created using the Consolidated Framework for Implementation Research (CFIR) [15]. Compared to other frameworks, such as the Theoretical Domains Framework (TDF), which focuses on behavior change at an individual level [16], CFIR was chosen to elicit more organization‐level constructs for interprofessional behavior change that could be implemented from our results. Interview questions elicited perspectives in four domains: (1) acceptability of efforts to standardize labor induction, (2) barriers and facilitators to implementation of standardized labor induction practices generally, (3) acceptability of, as well as barriers and facilitators to, implementation of specific components of the labor induction protocol, and (4) acceptability of current implementation strategies and suggestions for improved implementation.

This manuscript focuses on the third domain: clinician perspectives on individual components of the protocol. We aimed to examine how clinician perspectives differed among induction interventions and what unique barriers impacted different components. Components addressed in interviews focused on those with lower quantitative acceptability: (1) regular cervical examinations to assess for labor progress: every 2–4 h in latent labor and every 1–2 h in active labor, (2) amniotic membrane rupture if membranes were still intact and the cervix was ≥4 cm dilated, if clinically feasible, (3) hospital‐based oxytocin protocol (2 units/min, increasing by 2 units/min every 15 min as indicated, until a maximum dose of 40 units/min), and (4) IUPC placement if active labor was not progressing. For each component, clinicians were asked about their general beliefs, barriers to implementation, strategies they had utilized or seen to overcome component‐specific barriers, and their perception of the patient response to that component. A summary of interview questions specific to domain 3 is included in Table 1.

**TABLE 1:** Summary of interview questions addressing specific protocol components (domain 3).

Protocol component	Questions per component
*As described, the protocol contained multiple elements. I would like to know more about your experiences with and opinions about each. Specifically, I'd like you to reflect on the ease or difficulty of using each element, how frequently you used it (general ballpark is fine), barriers to using it (if any), how patients responded, and any strategies you employed to overcome barriers*.	
Frequency of cervical exams Rupture of membranes at 4 cm Use of oxytocin Placement of intrauterine pressure catheters	How easy or difficult do you find it to use/implement [component]? How frequently are you able to use/implement [component]? What barriers have you experienced with [component]? What do you tell patients about [component]? How do those conversations go or how do they respond? What strategies have you used to overcome barriers to [component]?

Semi‐structured interviews were conducted in‐person, via video conferencing software, or over the phone and lasted an average of 30 min. The primary investigator (R.F.H.), an obstetrician trained in qualitative interviewing, conducted all interviews. All interviews were recorded after consent was obtained. Audio from the interviews was transcribed by Datagain Transcription Services (Secaucus, NJ). Transcripts were then analyzed and coded using NVivo 12 software. A research coordinator trained in qualitative methods used an inductive process of iterative coding to develop a codebook by identifying recurrent relationships, themes, and categories. An integrated analysis approach [17] was then used, combining identification of a priori attributes and a modified content analysis approach. Two trained research personnel applied the codebook to the transcripts and periodically refined the themes and definitions based on inter‐rater reliability tests in order to facilitate analysis. A total of 20% of transcripts were double‐coded (*k*  =  0.83). Research coordinators then synthesized coding outputs and identified the key themes described in this manuscript.

## 3 RESULTS

A total of 104 clinicians (57 physicians, 43 RNs, 4 CNMs) completed the survey, and a total of 24 interviews were performed. Demographic characteristics of the participants are shown in Table 2.

**TABLE 2:** Demographic characteristics of participants. (1) Survey participants overall and (2) qualitative participants by acceptability group.

	Survey (quantitative) participants (*n* = 104)	High Acceptability qualitative participants (*n* = 12)	Low Acceptability qualitative participants (*n* = 12)
Role			
Attending physician	30 (28.8)	3 (25.0)	4 (33.3)
Resident/fellow physician	27 (26.0)	6 (50.0)	2 (16.7)
Certified nurse‐midwife	4 (3.8)	0 (0)	2 (16.7)
RN	43 (41.3)	3 (25.0)	4 (33.3)
Age			
≤35	57 (54.8)	7 (58.3)	7 (58.3)
36–45	34 (32.7)	4 (33.3)	1 (8.3)
46–55	9 (8.7)	1 (8.3)	2 (16.7)
≥56	4 (3.8)	0 (0)	2 (16.7)
Gender			
Female	91 (87.5)	11 (91.7)	11 (91.7)
Male	13 (12.5)	1 (8.3)	1 (8.3)
Years experience in role			
≤5	45 (43.3)	6 (50.0)	5 (41.7)
6–10	28 (27.0)	4 (33.3)	3 (25.0)
11–20	22 (21.2)	1 (8.3)	3 (25.0)
>20	9 (8.7)	1 (8.3)	1 (8.3)

### 3.1 Quantitative results

Median total AIM score across participants was 15/20 [IQR 12–19]. Quantitative data on overall protocol acceptability are summarized elsewhere [10]. Clinician comfort with individual protocol components, described as proportion of respondents who responded either “comfortable” or “completely comfortable” on the Likert scale for that component, is shown in Table 3. Protocol components with scores >70% comfort included discontinuation of futile cervical ripening (i.e., Foley balloon at 12 h, misoprostol at 6 doses or 24 h) and initiation of oxytocin, and initiation of oxytocin with unchanged cervical exams in active labor. Components with <70% comfort included frequent cervical exams, amniotomy at ≥4 cm, initiation of oxytocin if >6 h after last misoprostol dose, and IUPC placement with unchanged cervical exams on oxytocin in active labor.

**TABLE 3:** Induction protocol components with associated quantitative data.

Protocol component	% “comfortable”^a^ *N*(%)
1. If Foley balloon did not expel prior to 12 h, remove it and initiate/continue oxytocin.	72 (72.3)
2. Misoprostol can be repeated for up to 6 times and for no more than 24 h. If the patient remains in latent labor, initiate oxytocin.	73 (73.7)
3. If it has been >6 h since misoprostol placement, and artificial rupture of membranes (AROM) not yet feasible with no window for another misoprostol, start oxytocin.	56 (57.7)
4. Latent labor exams at least every 4 h.	43 (43.9)
5. If patient is ≥4 cm dilated and has intact membranes, recommend amniotomy.	41 (41.4)
6. Active labor exams at least every 2 h.	49 (50.0)
7. If two exams are the same in active labor 2 h apart and already s/p artificial or spontaneous rupture of membranes (AROM or SROM) but not on oxytocin, start oxytocin.	73 (74.5)
8. If two exams are the same in active labor 2 h apart and already s/p A/SROM and on oxytocin without IUPC in place, place IUPC.	62 (63.3)

^a^ “Comfortable” or “completely comfortable” with this recommendation on the Likert scale.

### 3.2 Qualitative results

A total of 24 interviews were performed, including 15 physicians (8 trainees and 7 attending‐level; 13 obstetrician‐gynecologists and 2 family medicine physicians), 2 certified nurse‐midwives, and 7 registered nurses. Of those interviewed, 12 participants were from the High Acceptability and 12 from the Low Acceptability groups. Median total AIM score was 20/20 [IQR 20–20] in the High and 12.5/20 [IQR 11–13] in the Low Acceptability groups. Demographic characteristics of qualitative participants are shown in Table 2. Qualitative data regarding acceptability of the protocol overall are addressed previously [10]. Additional quotations supporting component themes are presented in Table 4.

**TABLE 4:** Additional interview quotations addressing acceptability and barriers to use of individual labor induction protocol components.

Protocol component	Additional quotations
Frequent cervical exams	“I think in general, I think it's very easily utilized. I know patients like to know their progress, those time intervals.” (Nurse) “I feel like sometimes the nurse doesn't want us to check them that frequently in active labor or sometimes even in latent labor because they don't think it will change management at all. And so sometimes I'll have to sort of like talk to them why I'm doing that check.” (OB‐GYN resident physician) “I think we've set up expectations for patients who are in the earlier part of labor that if there's not a change in four hours, somehow, they're failing, it's not working.” (Nurse) “I think that patients are usually fine with the frequency of exams. And it's usually moderated in my experience by … discomfort during exam. So if someone has an epidural, and they're fine, or cervical checks have been comfortable for them, I haven't found folks particularly bothered about it. It's more when they're painful for the patient … which would be a minority of patients.” (Family Medicine resident physician)
Artificial rupture of membranes (AROM)	“I think that there are quite a few patients who don't want to get their water broken, either because of what they've heard before or they find it unnatural or they think it's going to hurt, all these various different reasons. So I do thing that it's definitely the step in the process that I think gets the most pushback.” (OB‐GYN resident physician) “I feel like it's great if it works and it oftentimes does, but I just feel like, again, you have to be sure. I have had many, many docs ask me for fundal pressure to do an AROM and I'm like, I don't think we need to do that that way, let's wait a bit, but again, I feel confident enough to say that and not every nurse does.” (Nurse)
Use of oxytocin	“[This piece of the protocol] has several challenges. One, is the myth that Pitocin [oxytocin] causes labor to be more painful, which I don't know how we can convince patients if that's not the case, but there is this kind of perpetuated fear of Pitocin in the community.” (OB‐GYN attending physician)
Placement of intrauterine pressure catheters (IUPCs)	“I find it that pretty easy to follow. I think sometimes just with education with our residents to make sure that they know that that is the plan and to follow the protocol should the exam be unchanged.” (Family Medicine attending physician)

#### 3.2.1 Frequent cervical examination

The standardized labor induction protocol recommended cervical exams every 4 h in latent labor and every 2 h in active labor. Participants generally felt these recommendations were easy to accomplish and were a helpful reminder to check in on patients, especially in active labor, a sentiment agreed upon by care team members.

However, a few participants felt that exams every 4 h were too frequent in latent labor, with concerns that (1) insufficient explanation was provided for the exam frequency, (2) nurses needed more time to help progress labor in between exams, and (3) that patients may develop inappropriate expectations about how much labor should progress between exams at this frequency.

As for barriers to implementation, many participants identified a lack of simultaneous physician/midwife and nursing availability as a challenge.

“The only time I feel that we run into issues with achieving those timelines is when the unit is very busy and there's not enough providers to be able to get to every patient at those time intervals.” (Nurse)

Others discussed a lack of consensus among the team about performing exams at the recommended frequency, especially if the patient made cervical change at the last check.

Strategies for overcoming these barriers included a team approach to performing exams, with other clinicians willing to jump in for a patient they weren't primarily managing when the primary clinician was occupied. Furthermore, inter‐team communication about when the next exam will be and its purpose can help minimize concerns about unnecessary interventions.

“If I feel like we're straying from the protocol or there's pushback on any aspect of that, I find it helpful to just regroup with the care team about why we're doing that exam… [to help] people understand the relevance and importance of doing exams at that time.” (Maternal Fetal Medicine fellow)

From the patient perspective, some participants mentioned how patients could influence variation from protocol recommendations such as requesting more frequent exams or deferring exams due to discomfort. A few participants discussed pain control methods for patients who found exams physically uncomfortable, including helping the patient de‐stress, changing physical position, or considering pain medications.

#### 3.2.2 Artificial rupture of membranes

The labor induction protocol recommends AROM or amniotomy at 4 cm dilation, if not previously ruptured and if feasible. Participants tended to feel more mixed about this protocol step. Many reported feeling the AROM recommendation was easy to follow and that they felt comfortable doing so, but multiple participants found this protocol step more difficult to implement than others.

The first barrier discussed was clinician resistance. Clinicians reported concerns about the strength of evidence, citing worries about risk of infection, fetal heart rate decelerations, or cesarean delivery.

“I think the biggest barrier is nursing pushback. Nurses are often concerned about the risk of infection, even though our studies show that AROM when appropriate overall reduces the time in labor without an associated risk of cesarean.” (OB‐GYN resident physician)

“I think that I personally struggle with that implementation… I'm somebody who's very supportive of a more natural, less active management of labor… I do think that subjectively a rupture of a primigravida without active labor can sometimes lead to higher incidence of variable [decelerations], chorioamnionitis, potentially C‐section… I think that if they're not actively laboring to rupture them, it could potentially cause more problems than good, and I'm not sure that we as an institution do a good job of explaining the risks of that to patients when we're rupturing them.” (Nurse)

Others described practical challenges such as less‐experienced clinicians’ difficulty determining feasibility of amniotomy and trainees’ fear of causing a cord to prolapse. Other practical barriers included need for frequent temperature checks once AROM was performed. Finally, patient hesitance made it difficult to adhere to this protocol recommendation, with some patients finding AROM too invasive or having preconceived notions around labor discomfort or need for regional anesthesia after AROM.

“I find this to be the most difficult aspect of the protocol to follow… patients and sometimes nurses have strong opinions about this. I think patients feel like it's a big step… Out of all the things we do, I think that [AROM] feels maybe most invasive to a patient or like it's a turning point in their labor induction.” (OB‐GYN attending physician)

When addressing these barriers, participants emphasized the importance of presenting evidence in favor of amniotomy to both patients and care team members, as well as clear education around its role in the labor induction protocol.

#### 3.2.3 Use of oxytocin

Oxytocin initiation is recommended by the protocol if 6 h have passed since the patient's last dose of misoprostol and the patient is contracting too much for additional misoprostol, or if patients have an unchanged exam over 2 h in active labor with already ruptured membranes. Once oxytocin administration has begun, the protocol recommends regular up‐titration. Participant reactions were typically positive to oxytocin use. Some felt that this step aligned well with their standard practice and that oxytocin was easy to use and/or they felt comfortable with doing so. Some nurses reported feeling more comfortable with oxytocin compared to other interventions due to its ability to be titrated and reversed.

However, others discussed skepticism around the strength of evidence supporting oxytocin and subsequent difficulty with implementing the protocol's recommendations:

“I think there's definitely nurse resistance. I mean I can say on my part, I don't feel like [the protocol‐recommended oxytocin use] has been proven to me.” (Nurse)

Other concerns were around practicality of up‐titrating oxytocin every 15 min, resulting in conflict within the team. Physician participants discussed challenges with nurses not consistently up‐titrating oxytocin and the need to repeatedly ask or remind nurses to do so. On the other hand, nurses reported labor floor acuity and fetal heart rate concerns as barriers to regular up‐titration.

Multiple strategies to overcome these barriers were discussed. Clinicians emphasized nurse education about up‐titration safety and tools to empower nurses to lead up‐titration. Recommendations for improved communication between team members about when oxytocin was not being regularly up‐titrated were also stressed by participants across roles.

“The only barrier to regular up‐titration other than unit acuity is sometimes with certain tracings, nurses aren't as comfortable increasing every 15 minutes… there needs to be conversations with providers that you're [waiting longer] and not just do it sort of on your own, just so that everybody's on the same page.” (Nurse)

Clinicians of different roles also reported facing patient resistance to oxytocin—occasionally, some patients feared the increased pain with contractions associated with starting oxytocin, but were typically receptive to the intervention after provider explanation.

#### 3.2.4 Placement of IUPCs

Placement of an IUPC is recommended by the protocol when the patient's cervical dilation has been the same in active labor more than 3 h apart, if the patient's membranes have already been ruptured and they are already on oxytocin. Most participants expressed positive sentiment about this step, with some citing patient‐specific factors that could impact their enthusiasm around IUPC use such as whether patients had an epidural.

“I think it is generally a good thing. I think it's good to know how strong your contractions are and I think it can be useful… [But] there's a certain amount of individuality to how labor works and… I don't like to see everybody getting one because of two exams.” (Nurse)

While some participants felt this step was easy to implement, some reported challenges such as differences in provider familiarity with IUPC as the next clinical step in the protocol. For example, some discussed that trainees may want confirmation that it is safe to place the catheter and may therefore defer placement. Others discussed clinician resistance or misinformation around the appropriate clinical situation for placement, particularly from nursing colleagues.

From a patient's perspective, participants reported that patients occasionally feared that the catheter would hurt their baby, but were overall more receptive after a provider explained their functionality and low risk. Participants emphasized the need for nurse and patient education about IUPC safety, including explaining their role in the protocol, as a strategy for avoiding cesareans, their low risk of complication, and their ability to help monitor labor progress.

“The education piece with the patient… a lot of times they don't understand what that means for them and that there's something that's going inside of their body to measure the contractions, but once the education piece is put out there usually, there's not a lot of concern.” (Nurse)

## 4 DISCUSSION AND CONCLUSION

Standardization of labor induction has been suggested as a method to improve obstetric care equity, as well as maternal and neonatal outcomes. Yet, the success of such protocols is limited by implementation challenges unique to each protocol component. This work expands on a previous study of the clinician perspective on standardizing labor induction by exploring perspectives on individual components of a labor induction protocol from both a quantitative and qualitative approach, with specific recommendations for cervical exam frequency, timing of amniotomy, initiation and up‐titration of oxytocin, and use of IUPCs. Overall, the most acceptable components focused on not continuing “futile” cervical ripening, as well as interventions for active labor dystocia, while clinicians were more hesitant about frequent cervical exams or early amniotomy. In particular, this work characterizes barriers to implementation of each of these protocol steps and details suggested strategies to overcome them.

While there were some barriers unique to specific components, this study demonstrated several overarching themes. Clinician‐perceived barriers to protocol adherence included (1) floor census and acuity, (2) resistance among clinicians of other roles, and (3) patient resistance to interventions. Across all protocol components, the importance of increased education for both clinicians and patients on protocol components’ safety and evidence for advancing labor was highlighted. For patients, this education could occur before presenting for induction to ensure patients are thoroughly informed about the process. Protocols for labor induction could not only focus on shared decision‐making during labor itself, but also on standardized patient induction education in the outpatient setting. In parallel, interdisciplinary, formal clinician education could be paired with standardized reference materials on protocol steps. Another key strategy identified was prioritizing communication between team members and patients to facilitate protocol implementation. For example, discussions between the physician or midwife, nurse, and patient after each check could empower all team members to advocate for the step to be performed at the appropriate time. These recommendations are summarized in Table 5.

**TABLE 5:** Summary of the clinician perspective on barriers to protocol implementation and suggested strategies to overcome each.

Barrier	Recommended strategies to overcome barriers
Floor census and acuity	Frequent communication among team members about availability, timing of next steps. Record planned next step and timing in a public place for all clinician types to be aware. Collaboration across multiple providers and nurses to advance care. Encourage a culture of jumping in to help perform induction steps when appropriate rather than waiting for a specific clinician when the floor is busy.
Clinicians encountering resistance among clinicians of other roles	Increased, formal education around the evidence surrounding specific interventions (e.g., amniotomy, oxytocin, IUPCs) and their safety, efficacy, and role in labor induction; education can be through department meetings, huddles, and/or virtual trainings, but must be multidisciplinary and cyclical due to nursing and clinician turnover. Interdisciplinary, standardized education is needed to ensure clinicians of all roles are able to engage in conversation around the evidence and share expertise, as different clinicians may have different interpretations or insights on existing evidence. Interdisciplinary communication and open conversation around anticipated next steps. Some ways to do this include a discussion between the clinician, nurse, and patient about the timing of the next step prior to leaving the patient's room. Empower all clinicians to request next protocol step be performed. Printed materials for clinicians experiencing resistance from other clinicians to refer to regarding the next steps in the protocol.
Patient resistance to interventions	Education around the safety, efficacy, and role of the overall induction protocol, as well as individual interventions. This education can be done outpatient when scheduling inductions through written or video‐based education in addition to interactive clinician counseling. Open, two‐way dialogue around labor course and next steps; consider signs showing patients the protocol and encourage them to ask about next steps.

Although clinicians have generally reported positive sentiment toward standardizing labor induction [10], this study expands this analysis to compare clinician opinions on different protocol components and identify ways to improve implementation of a standardized protocol, the first study to report on such perspectives. The need for such work is driven by emerging evidence that standardizing labor induction can improve obstetric outcomes and decrease racial disparities [2, 3, 4, 5, 6, 7, 8]. The themes identified around the clinician–patient relationship also align with qualitative literature on patient labor experiences that corroborates the importance of patient education and clear communication to allow for shared decision‐making [18, 19]. Finally, while this study specifically focuses on labor induction, the themes identified here can be globally applicable to labor management outside of an induction setting as well. Interdisciplinary communication and more integrated provider and patient education are critical to labor management in all settings.

Strengths of this study include its mixed‐methods approach and implementation science frameworks, with targeted goals of developing concrete recommendations for future implementation. Our study also included clinicians in diverse roles, with qualitative data collected from the most data‐rich individuals. Limitations include that our study analyzes one specific protocol, potentially limiting its generalizability. We were also not powered to compare acceptability by clinician role or by clinical site. Finally, both of our surveyed clinical sites, though different in practice model and patient acuity, represent academic urban health centers in a single city and may not allow for generalizability to other practice settings and geographic regions; the unique hierarchical structure, prevalence of trainees, and dedicated in‐house clinical teams at academic institutions may differ significantly from community or rural hospital settings.

Future research to build on the findings of this study should analyze the impact of incorporating the proposed strategies for improving implementation of a labor induction protocol. Further analyses with larger cohorts can evaluate predictors of clinician acceptability, such as by role or clinical site, allowing for more focused implementation strategies targeted toward specific clinicians and practice models.

## CONFLICT OF INTEREST STATEMENT

The authors declare no conflicts of interest.

## Data Availability

The data that support the findings of this study are available from the corresponding author upon reasonable request.
